# Representational deficit or processing effect? An electrophysiological study of noun-noun compound processing by very advanced L2 speakers of English

**DOI:** 10.3389/fpsyg.2015.00077

**Published:** 2015-02-09

**Authors:** Cecile De Cat, Ekaterini Klepousniotou, R. Harald Baayen

**Affiliations:** ^1^Department of Linguistics and Phonetics, University of LeedsLeeds, UK; ^2^School of Psychology, University of LeedsLeeds, UK; ^3^Quantitative Linguistics Lab, Department of Linguistics, Eberhard Karls University TübingenTübingen, Germany

**Keywords:** compounds, second language, word order, ERP, frequency effects, generalized additive mixed models

## Abstract

The processing of English noun-noun compounds (NNCs) was investigated to identify the extent and nature of differences between the performance of native speakers of English and advanced Spanish and German non-native speakers of English. The study sought to establish whether the word order of the equivalent structure in the non-native speakers' mothertongue (L1) had an influence on their processing of NNCs in their second language (L2), and whether this influence was due to differences in grammatical representation (i.e., incomplete acquisition of the relevant structure) or processing effects. Two mask-primed lexical decision experiments were conducted in which compounds were presented with their constituent nouns in licit vs. reversed order. The first experiment used a speeded lexical decision task with reaction time registration, and the second a delayed lexical decision task with EEG registration. There were no significant group differences in accuracy in the licit word order condition, suggesting that the grammatical representation had been fully acquired by the non-native speakers. However, the Spanish speakers made slightly more errors with the reversed order and had longer response times, suggesting an L1 interference effect (as the reverse order matches the licit word order in Spanish). The EEG data, analyzed with generalized additive mixed models, further supported this hypothesis. The EEG waveform of the non-native speakers was characterized by a slightly later onset N400 in the violation condition (reversed constituent order). Compound frequency predicted the amplitude of the EEG signal for the licit word order for native speakers, but for the reversed constituent order for Spanish speakers—the licit order in their L1—supporting the hypothesis that Spanish speakers are affected by interferences from their L1. The pattern of results for the German speakers in the violation condition suggested a strong conflict arising due to licit constituents being presented in an order that conflicts with the expected order in both their L1 and L2.

## 1. Introduction

Noun-noun compounds are entities consisting in two nouns united by a semantic relation (Gagné and Spalding, 2014) that is not overtly expressed. Endocentric compounds contain a head element (*dust* in 1) whose lexical category and interpretive features are inherited by the compound and contribute the core of its meaning (e.g., a kind of dust). The other element acts as a modifier of that head.

(1) moon dust (“dust from the moon” / “dust made of moon” / “dust with moon-like properties”)

Compounds have been extensively studied in the past 40 years from a myriad of viewpoints (Libben and Jarema, 2006; Lieber and Štekauer, 2009; Semenza and Luzzatti, 2014). A key concern has been whether the processing of compounds consists of retrieving entities listed in the mind (Butterworth, 1983) or requires decomposition into constituents listed separately (Semenza et al., 1997; Libben, 1998). Dual-route theories contend that the two processes (i.e., a whole-word and a parsing procedure) exist side by side (Sandra, 1990). It is now widely accepted that both constituents are activated during processing, at least in non-lexicalised compounds (Jarema, 2006; Zhang et al., 2012; MacGregor and Shtyrov, 2013). Noun-noun compounds have also been shown to be processed differently to non-compounds of similar morphological complexity and length, with compounds yielding longer reaction times and different electrophysiological correlates (El Yagoubi et al., 2008).

Here we focus on endocentric noun-noun compounds (henceforth NNCs), which have been argued to embody an underlying structure (Libben, 2006): their structure is hierarchical, involving the (possibly recursive) subordination of a modifier to a grammatical head (or a modifier-head compound, as in 2-b).

(2) a. [[[lunch box] lid] stack]
    b. [ child[ amateur [puppet theater]]]

These characteristics suggest that NNCs involve phrasal syntax. Diachronic and synchronic corroborating evidence is provided by (Zipser, 2013): cross-linguistically, (i) the constituent order of compounds reflects the current word order or an earlier word order found in the underlying phrases (e.g., *nut-cracker* shows SOV, the Old English word order); (ii) adjective-noun compounds are not recursive, as predicted by the fact that adjectives do not allow adjective complements; and (iii) recursive compounding is possible only in right-branching phrase structures.

What makes the acquisition of NNCs by non-native speakers particularly interesting to study is that the syntactic properties they exhibit (hierarchical structure, head directionality) are predicted to be acquired very early^1^, and their interpretation is essentially a matter of phrasal semantics (which has been shown not to cause persistent difficulty for L2 learners, see Slabakova, 2008). NNCs also appear very early in L1 acquisition (Nicoladis and Yin, 2002; Krott et al., 2010). All this predicts that the processing of NNCs should be relatively unproblematic for advanced learners of English. In particular, L1 word-order effects are not expected: L2ers whose L1 features the opposite word order (i.e., head-first) should not accept English NNCs in reversed order more than L2ers whose L1 order matches that of English. At an advanced level of proficiency, both groups are expected to reject irreversible compounds presented in reversed order:

(3) a. #[ [ basket ] dog ] → uninterpretable as head-last
    b. ^*^[ basket [ dog ] ] → head-first order is ungrammatical

Headedness plays a specific role in the processing of NNCs, as shown by research on Italian (which features the two word orders in NNCs): based on a lexical decision task on healthy adults, (El Yagoubi et al., 2008) found clear effects induced by the head, independently of its position in the NNC. Arcara et al. (2014) recently argued that (in Italian) NNCs are decomposed differently, depending on whether they are head-initial or head-final, the latter requiring a higher processing effort when decomposition is elicited. This suggests that in Italian, only head-final compounds are true hierarchical structures (as opposed to lexicalised syntactic units)—see Marelli et al. (2009, 2014). Headedness effects are not distinguishable from position-in-the-string effects in languages such as English. For instance, Jarema et al. (1999) observed no difference in the priming of NNCs by the head or the modifier. This paper takes this line of research further, by investigating whether L1 headedness affects the L2 processing of transparent, irreversible NNCs in very advanced learners of English. In two separate studies, we examined the reaction times and the event-related potentials in response to irreversible NNCs presented in licit vs. reversed word order.

Event-related potentials (ERPs) can provide insight into the neural activity associated with the processing of compounds. Functional interpretations can be inferred from the temporal and spatial characteristics of electromagnetic activity, and ERP components can sometimes reveal the engagement of the cognitive processes involved. Our approach in this paper is exploratory (Otten and Rugg, 2005) and will focus on identifying differences in the amplitude of the EEG signal that can be traced back to properties of the participants (such as their language background) and properties of the compounds (such as their frequency of occurrence, and the frequencies of occurrence of their constituents). Inferences based on previously identified ERP components will be drawn in the discussion as appropriate.

Our research questions are: (i) Does non-native processing of NNCs result in different ERP signatures to native processing? (ii) Is non-native processing of NNCs affected by headedness effects from the mother tongue?

We hypothesize that, if very advanced L2 learners are affected by their L1's headedness settings (in spite of the early parameter resetting), the performance of L2ers whose L1 displays the same word order as English (here: German) will be different to that of those whose L1 doesn't (here: Spanish). A significant proportion of erroneous judgements would be taken to indicate a representational deficit (i.e., incomplete acquisition of the target structure). Longer reaction times are expected for both L2 groups, in line with much research on L2 processing (Kroll et al., 2002; Moreno and Kutas, 2005; Clahsen et al., 2013), but significantly longer reaction times in the Spanish group than in the German group would indicate a specific L1 effect. Differences in the processing mechanisms themselves should translate into significantly different ERP signatures across participant groups.

Furthermore, following up on research on compound processing with eye-movement registration (see e.g., Hyönä and Pollatsek, 1998; Pollatsek et al., 2000; Juhasz et al., 2003; Bertram et al., 2004; Kuperman et al., 2008, 2009; Miwa et al., 2014), we expected compound and constituent frequency as covariates to offer enhanced insights into how German and Spanish advanced learners of English differ from native speakers of English when presented with English compounds with constituents presented in the standard as well as in the reversed order. More specifically, we expected that compound frequency, if useful as a predictor, should modulate the EEG amplitude primarily for native speakers, given that less proficient readers have been observed to show decompositional eye-movement patterns (see Kuperman and Van Dyke, 2011, for English). In addition, constituent frequency effects, ubiquitous in the behavioral and eye-tracking literature, should also be detectable. Since compounds with constituents presented in reversed order can only be made sense of by interpreting the constituents, we expected the strongest constituent effects to be present in the reversed condition.

## 2. Materials and methods

In order to assess whether any L1 headedness effect affects L2ers' processing of NNCs, we carried out two separate studies based on the same task. We registered the accuracy and (i) the timed response or (ii) the electrophysiological response of the brain to visual stimuli presented in the context of a primed lexical decision task. Stimuli were irreversible NNCs presented in licit (4-a) and reversed order (4-b).

(4) a. coal dust
    b. #dust coal

The participant groups differed in mother tongue: English (control group), Spanish or German (experimental groups). Like English, German features productive compounding, with a head-last structure (Meyer, 1993). Whereas in Spanish, compounds are essentially head-first, and not productive (Piera, 1995).

### 2.1. Participants

Ten native British English speakers, ten native German learners of English and ten native Spanish learners of English took part in each study (i.e., a different group in each study, as detailed in Table 1). Non-native participants all had initial second-language exposure after 8 years of age, and all scored above 60% on a cloze test from the Cambridge Certificate in Advanced English. All participants were right-handed based on the Briggs and Nebes inventory (Briggs and Nebes, 1975), had no speech or language difficulties and had normal or corrected-to normal vision. Ethical approval was issued by the School of Psychology, University of Leeds, and informed written consent was obtained from all volunteers.

**Table 1:** **Participant characteristics**.

**L1**	**English**	**German**	**Spanish**
**STUDY 1**			
Female/Male	7/3	8/2	5/4
Mean age (+ SD)	23;8 (3;10)	23;2 (0;11)	25;4 (6;10)
Mean proficiency (+ SD)		75% (13)	80% (9)
**STUDY 2**			
Female/Male	4/6	7/3	3/7
Mean age (+ SD)	22;11 (3;3)	26;5 (5;7)	26;11 (5;3)
Mean proficiency (+ SD)		90% (7)	81% (8)

### 2.2. Stimuli

Experimental stimuli consisted of prime-target pairs, presented in 4 experimental conditions in a 3 (Group) × 2 (Prime Condition) × 2 (Word Order) design. The prime was either the head (e.g., *dust* in 4) or the modifier (e.g., *coal* in 4) of the intended compound.

The Word Order factor had 2 levels: licit (modifier - head, as in 4-a) or reversed (head—modifier, as in 4-b). All the NNCs were endocentric and featured a transparent modification relationship. All items were tested for irreversibility on an independent group of 30 native speakers^2^.

The frequency of the licit compounds and their constituent nouns was estimated from the post-1990 data in Google N-grams. To avoid lexicalisation effects, only compounds with very low frequencies were included (i.e., below 3300—mean = 359.5, compared with a mean of 279,300 for the constituent nouns).

There was a total of 480 test items (based on 120 compounds), of which 234 are included in the present study (as we focus on the Head Prime condition only, and 3 compounds had to be discarded due to spelling inconsistencies between the licit and the reversed word order conditions). All the compounds were with spaced constituents. The items were pseudo-randomized into 8 different orders (assigned randomly to participants) and presented in 4 blocks, with a rest in between^3^.

## 3. Study 1: primed lexical decision

### 3.1. Procedure

Participants were tested individually in a single session lasting approximately 20 min. Stimuli were presented visually in light gray text on a black background. Each trial began with a 100 ms mask (#######), after which the prime was presented for 100 ms followed by a second mask (for 50 ms) and the target (for 8000 ms). Participants had to make a lexical decision about the target (as acceptable or not) by pressing (with their right hand) one of two buttons on a hand-held button box (counterbalanced across participants). We recorded accuracy rates and reaction times from the onset of presentation of the target, using E-Prime software.

### 3.2. Results

Only responses whose reaction times fell between 150 and 5000 ms were included in the analysis, on the assumption that faster responses would not allow sufficient processing time to yield an acceptability judgment, and slower responses are likely to result from conscious processes (0.003% of data were thus excluded). One Spanish participant was excluded due to production of 40% of the responses above the 5000 ms threshold and borderline proficiency given our inclusion criteria.

As seen in Table 1 (after exclusion of the abovementioned participant), the proficiency of the Spanish group was slightly higher than that of the German group (Wilcoxon rank sum test: *W* = 2049133, *p* < 0.0001).

#### 3.2.1. Accuracy analysis

Table 2 shows that accuracy was very high overall in all groups, and that the predominant type of error was to accept compounds in the reversed order (rather than reject licit compounds).

**Table 2:** **Proportions and types of errors across groups in Study 1**.

	**English**	**German**	**Spanish**
Accept reversed	5.97	10.29	14.67
Reject licit	3.98	6.77	6.64
Correct	90.06	82.94	78.69

The responses on the lexical decision task were analyzed with a generalized linear mixed-effect model with a logit link function and binomial variance, using the lme4 package, version 1.0-4 (Bates et al., 2013) with the “bobyqa” optimizer, using treatment dummy coding for factorial predictors. Only those predictors that contributed to the model fit were retained, as shown in Table 3. As a consequence, the frequency covariates, which did not reach significance, were removed from the model specification. The resulting model provided a substantially improved fit compared to the null-hypothesis model with random intercepts for participant and item only (and with random slopes for word order condition by participant, and participant group by item)^4^.

**Table 3:** **Coefficients of a logistic mixed-effects regression model fitted to the accuracy data of Study 1, and associated statistics**.

	**Coefficient**	**Std. Error**	**Z**	**p**
Intercept	−0.1960	1.0842	−0.1808	0.8565
Word.Order:Reversed	−0.4439	0.2382	−1.8639	0.0623
L1: German	0.0344	0.3751	0.0917	0.9269
L1: Spanish	−0.0110	0.3491	−0.0316	0.9748
Proficiency	3.0819	1.0657	2.8920	0.0038
Word.Order:Reversed by L1: German	−0.1355	0.2743	−0.4938	0.6214
Word.Order:Reversed by L1: Spanish	−0.7004	0.2795	−2.5060	0.0122

*The reference level for Word Order is Licit, and for L1: English*.

**Figure 1:**
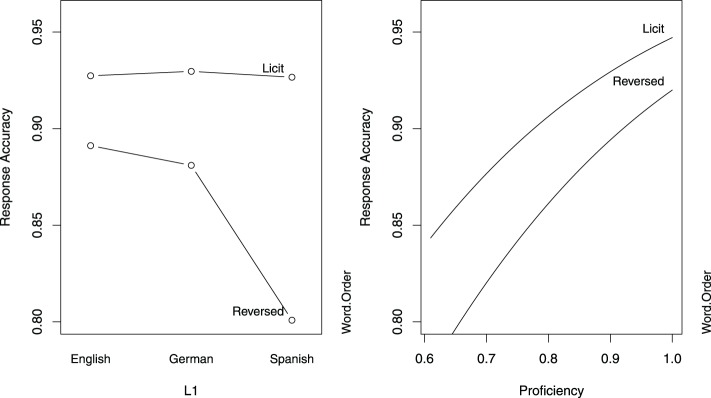
**Partial effects of the predictors in the logistic model for response accuracy in Study 1**. The left panel is calibrated for the reference levels of Word Order (Licit) and L1 (English), and the right panel is calibrated for median proficiency.

Table 3 indicates that for English speakers, accuracy was higher in the licit word order condition. Furthermore, in the licit word order condition, accuracy levels are comparable in native and non-native speakers, as can also be seen in the left panel of Figure 1. In the reversed word order condition, only the Spanish group performed significantly worse than the native speakers. Across groups, greater proficiency afforded higher accuracy, as illustrated in the right panel of Figure 1.

#### 3.2.2. Reaction times analysis

**Figure 2:**
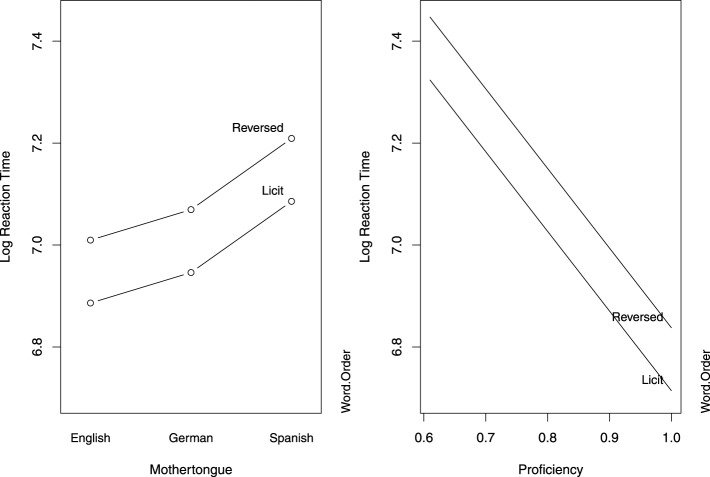
**Partial effects of the predictors in the mixed-effects model fit to the log-transformed response latencies in Study 1**. The left panel is calibrated for the reference levels of Word Order (Licit) and L1 (English), and the right panel is calibrated for median proficiency.

An analysis of the response latencies, summarized in Table 4 and visualized in Figure 2, indicated that all groups were faster at rating compounds in the licit word order condition. Only the Spanish group responded significantly slower than the English group. Speed increased with proficiency. The frequency measures did not reach significance nor improve the model fit, and were therefore removed from the final model^5^.

**Table 4:** **Coefficients of a logistic mixed-effects regression model fitted to the reaction time data**.

	**Coefficient**	**Std. Error**	***t*-value**
(Intercept)	8.2770	0.3100	26.7140
Word.Order:Reversed	0.1230	0.0180	6.7140
L1German	0.0600	0.1020	0.5840
L1Spanish	0.1990	0.0940	2.1160
Proficiency	−1.5620	0.3060	−5.1100

*The reference level for Word Order is Licit, and for L1, English. Absolute values of *t* exceeding 2 are indicative of significance at the 5% level*.

## 4. Study 2: event-related potentials with primed lexical decision

### 4.1. Procedure

The stimuli were the same as in Study 1, and participants were subject to the same inclusion criteria (see Table 1 for details).

Participants were tested individually in a single session lasting approximately one and a half hours. Stimuli were presented visually in light gray text on a black background. Each trial began with the visual presentation of a series of exclamation points (!!!) for 1000 ms, which was a signal for the participant to rest their eyes and blink. After a delay of 100 ms a fixation point (+) was presented for 250 ms to signal that the trial was about to begin and to alert participants that they had to fixate their eyes and avoid eye movements until the next set of exclamation points. A mask (#######) was then presented for 100 ms after which the prime was presented for 100 ms followed by a second mask (#######) for 50 ms and the target for 1000 ms^6^. After a delay of 500 ms a question mark (?) appeared for 2000 ms during which time participants had to make a lexical decision about the target (decide whether or not it was grammatical in English) by pressing one of two buttons on a hand held button box (counterbalanced across participants). Participants were instructed to respond as accurately as possible; accuracy and reaction times (in ms from the onset of the “?”) were recorded. (We do however not report on the reaction times below, as they reflected answer to the cue “?” rather than to the stimuli.) After the response (or at the end of 2000 ms if the participant did not respond), there was a delay of 100 ms before the next trial started. The experimental session was preceded by a practice session comprising 20 trials, which was repeated until participants could perform the task and procedure with no errors and no eye movements during the critical period of stimulus presentation (usually one or two practice sessions were required).

The EEG was recorded (Neuroscan Synamps2) from 60 Ag/AgCl electrodes embedded in a cap based on the extended version of the International 10–20 positioning system (Sharbrough et al., 1991) and fitted with QuikCell liquid electrolyte application system (Compumedics Neuroscan). Additional electrodes were placed on the left and right mastoids. Data were recorded using a central reference electrode placed between Cz and CPz. The ground electrode was positioned between Fz and FPz. To monitor eye movements, electro-oculograms (EOGs) were recorded using electrodes positioned at either side of the eyes, and above and below the left eye. At the beginning of the experiment electrode impedances were below 10 kΩ. The analog EEG and EOG recordings were amplified (band pass filter 0.1–100 Hz), and continuously digitized (32-bit) at a sampling frequency of 500 Hz.

Data were processed offline using Neuroscan Edit 4.3 software (Compumedics Neuroscan) and filtered (0.1–40 Hz, 96 dB/Oct, Butterworth zero phase filter), inspected visually and segments contaminated by muscular movement marked as bad. The effect of eye-blink artifacts was minimized by estimating and correcting their contribution to the EEG using a regression procedure which involves calculating an average blink from 32 blinks for each participant, and removing the contribution of the blink from all other channels on a point-by-point basis. Data were epoched between –100 and 1100 ms relative to the onset of the experimental targets and baseline-corrected by subtracting the mean amplitude over the pre-stimulus interval. Epochs were rejected if participants did not make a response within the allocated time (during presentation of the “?"), or if they made an incorrect response. Subsequently the data was downsampled to 125 Hz. Trial rejection was not done *a priori* but based on the residuals of the modeling, resulting in only 0.7% of discarded data.

### 4.2. Results

#### 4.2.1. Accuracy analysis

**Figure 3:**
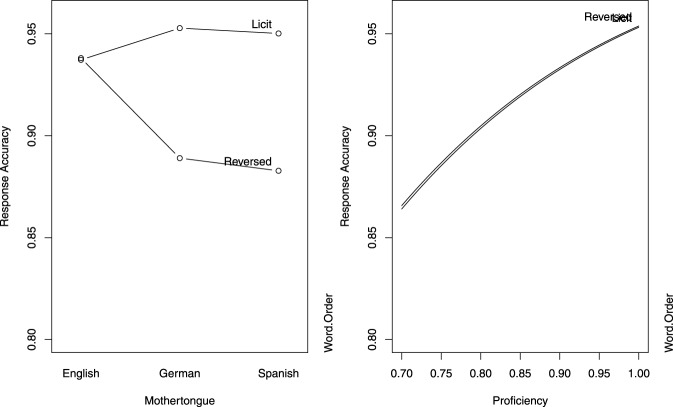
**Partial effects of the predictors in the logistic model for response accuracy in Study 2 (delayed primed lexical decision)**. The left panel is calibrated for the reference levels of Word Order (Licit) and L1 (English), and the right panel is calibrated for median proficiency.

A mixed-effects logistic regression model was fitted to the accuracy data. Results are summarized in Table 5, and displayed in Figure 3,^7^. For English speakers, accuracy did not differ significantly for the licit and reversed word order conditions. For both groups of non-native speakers, accuracy was higher in the Licit Word Order condition, compared with the Reversed Word Order condition. Across groups, greater proficiency afforded higher accuracy.

**Table 5:** **Coefficients of a logistic mixed-effects regression model fitted to the accuracy data**.

	**Coefficient**	**Std. Error**	**Z**	**p**
Intercept	−0.8676	1.7482	−0.4963	0.6197
Word.Order:Reversed	0.3013	0.4174	0.7218	0.4704
L1: German	0.2444	0.4981	0.4907	0.6236
L1: Spanish	0.0142	0.3072	0.0462	0.9631
Proficiency	3.8813	1.7261	2.2487	0.0245
Word.Order:Reversed by L1: German	−0.9381	0.4039	−2.3224	0.0202
Word.Order:Reversed by L1: Spanish	−0.9426	0.3999	−2.3571	0.0184

*The reference level for Word.Order is Licit, and for L1: English*.

The main difference in the pattern of results therefore concerns the effect of the word order manipulation, which adversely affected responses for English speakers in the reversed condition in “immediate” lexical decision, but had no consequences for English speakers in the delayed lexical decision task. In addition, when responses are delayed, German speakers pattern together with the Spanish speakers in their response behavior.

#### 4.2.2. ERP analysis

We include for analysis only trials that elicited a correct response. The time window analyzed was limited to 0–800 ms, time-locked to the onset of stimulus presentation^8^.

We analyzed the electrophysiological response elicited by the presentation of compound words with the generalized additive mixed model (gamm, Wood, 2004, 2006; Tremblay and Baayen, 2010; Kryuchkova et al., 2012; Tremblay and Newman, 2015; Baayen, in preparation; Baayen et al., in preparation). Generalized additive mixed models are a relatively novel extension to the generalized linear mixed model, and offer the analyst tools (such as thin plate regression splines and tensor product smooths) for modeling *non-linear* functional relations between one or more predictors and a response variable. This is essential for regression modeling of a response such as the amplitude of the EEG signal, which varies nonlinearly with time.

For regression modeling—which we will need to study the effect of compound frequency as well as compound constituent frequencies—GAMMs, as implemented in the mgcv package 1.7–28, offer the possibility of modeling the EEG amplitude as a nonlinear function of time and frequency simultaneously, resulting in potentially wiggly surfaces (or, in case of more than two numerical predictors, in wiggly hypersurfaces). By decomposing the EEG amplitude into a sequence of additive components, GAMMs afford the analyst a toolkit for separating out (potentially non-linear) partial effects due to different kinds of predictors (e.g., language group, time, compound frequency, constituent frequency).

In addition, GAMMs can capture AR1 autocorrelative processes in the signal, and therefore offer some protection against anti-conservative *p*-values and mistakingly taking noise for complex ERP signatures (as has been shown to occur by Tanner et al., 2013)^9^. For the present analysis, most autocorrelative structure in the residual error was removed by including in the gamm an autocorrelation parameter ρ = 0.9 for AR1 error for each basic time series in the data (the time series amplitudes for each unique combination of subject and item). Thanks to inclusion of the ρ parameter, there was little remaining autocorrelation in the model's residuals, as required.

Finally, we analyzed the EEG amplitude without any prior aggregation, seeking to predict the development of the EEG amplitude over time for any individual combination of subject and item. With 609,500 observations at each channel, we refrained from fitting a single GAMM to the full dataset. Instead, we fitted a separate GAMM to individual channels (i.e., the electrodes were analyzed independently), expecting to find similar regression curves and regression surfaces at neighboring channels. In other words, precisely because channels are not independent, topographical consistency can be relied upon as a criterion for having confidence in the regression effects.

The GAMMs provided by the mgcv package are designed to work fluently with treatment coding for factorial predictors. In order to inspect potential interactions between L1 group (three levels) and Word Order (two levels), we created a new six-level factor, which we labeled OG (“ordered grouping”), with levels English:Licit, English:Reversed, German:Licit, German:Reversed, Spanish:Licit, and Spanish:Reversed, with English:Licit as reference level.

Thus, we modeled the amplitude of the EEG signal (without any prior averaging) as an additive function of the fixed-effect factor OG and three covariates: Compound Frequency, and the Constituent Frequencies of Modifier and Head. Proficiency did not reach significance and did not improve the model fit significantly, so we did not include this covariate in the final model.

Participant and Compound were included in the model as random-effect factors. For Compound, we included random intercepts, in order to allow for differences in baseline amplitude across compounds. For Participant, we included two separate random-effects structures: a nonlinear factor smooth for Trial, and a second nonlinear factor smooth for Time. (These factor smooths are the non-linear counterpart of what in a strictly linear model would have to be modeled by the combination of random intercepts and random slopes, i.e., by-participant calibration of regression lines.) The factor smooths for Trial model the development of a subject's amplitude over the course of the experiment. The factor smooths for Time model a subject's typical development of the EEG amplitude while being exposed to a given compound. These factor smooths typically afford substantial improvement to the model fit, but as these smooths are not of theoretical interest in the framework of this study, we do not discuss them in detail.

Table 6 presents a summary of the GAMM fitted to the EEG amplitude at channel C3^10^. The upper half of this table presents the parametric part of the model, with coefficients familiar from standard linear modeling with treatment coding for factors. The first six rows present the intercept (representing the group mean for English speakers in the licit word order condition, for log-transformed compound and constituent frequencies equal to 0), and the changes in the intercept for the five other factor levels. The only significant difference pertains to English speakers responding to compounds with reversed word order. In this condition, the mean amplitude was shifted down by 0.64. The second six rows summarize the effect of (log) Compound Frequency, which turned out to be linear. For English speakers presented with compounds with normal constituent order, a greater compound frequency predicted lower-valued amplitudes. The differences in slope for the other five combinations of group and word order indicate that here the slopes for Compound Frequency were around zero. For instance, for the English Reversed condition, the slope was −0.14 + 0.17 = 0.03. A separate model (not shown) testing the six slopes against zero revealed, as expected, a significant negative slope for licit compounds in English, and also a reduced negative slope (−0.078) for reversed compound for Spanish speakers (*p* = 0.0414). Thus, the Spanish speakers show, for the reversed condition, a pattern that resembles, albeit in weakened form, the pattern observed for English in the licit condition. Recall that in the non-delayed lexical decision task (Study 1), Spanish speakers responded with reduced accuracy in the reversed condition, compared to English speakers. Since in Spanish, the reversed word order would be the licit order, we may be seeing in the EEG amplitude the consequences of expecting (given one's L1 experience) a given constituent order (the licit order for English, but the reversed order for Spanish speakers).

**Table 6:** **Generalized additive mixed model fitted to the amplitude of the electrophysiological response of the brain to English compounds at channel C3**.

**A. Parametric coefficients**	**Estimate**	**Std. Error**	***t*-value**	***p*-value**
Intercept (English licit)	0.5974	0.6793	0.8794	0.3792
Intercept Δ English reversed	−0.6369	0.1657	−3.8440	0.0001
Intercept Δ German licit	−1.2366	0.9314	−1.3276	0.1843
Intercept Δ German reversed	−1.5237	0.9320	−1.6348	0.1021
Intercept Δ Spanish licit	−0.2747	0.9336	−0.2942	0.7686
Intercept Δ Spanish reversed	0.6333	0.9322	0.6794	0.4969
Compound frequency (English licit)	−0.1385	0.0368	−3.7636	0.0002
Compound frequency: Δ English reversed	0.1731	0.0350	4.9499	<0.0001
Compound frequency: Δ German licit	0.1117	0.0352	3.1748	0.0015
Compound frequency: Δ German reversed	0.1154	0.0359	3.2122	0.0013
Compound frequency: Δ Spanish licit	0.1971	0.0354	5.5691	<0.0001
Compound frequency: Δ Spanish reversed	0.0606	0.0363	1.6685	0.0952
**B. Smooth terms**	**edf**	**Ref.df**	***F*-value**	***p*-value**
Spline smooth time (English licit)	8.5375	8.6981	12.3205	<0.0001
Spline smooth time: Δ English reversed	3.3899	4.3034	6.5872	<0.0001
Spline smooth time: Δ German licit	1.0013	1.0018	3.8845	0.0487
Spline smooth time: Δ German reversed	4.1062	5.1882	3.1005	0.0078
Spline smooth time: Δ Spanish licit	3.9976	5.0409	6.8602	<0.0001
Spline smooth time: Δ Spanish reversed	1.0227	1.0320	0.9527	0.3293
Tensor smooth freq C1, Freq C2 (English licit)	9.9401	10.6705	4.1504	<0.0001
Tensor smooth freq C1, Freq C2: Δ English:Reversed	7.4023	8.5028	4.6581	<0.0001
Tensor smooth freq C1, Freq C2: Δ German:Licit	11.7144	12.3939	8.8861	<0.0001
Tensor smooth freq C1, Freq C2: Δ German:Reversed	6.9721	8.1846	4.9458	<0.0001
Tensor smooth freq C1, Freq C2: Δ Spanish:Licit	9.4385	10.4868	11.6824	<0.0001
Tensor smooth freq C1, Freq C2: Δ Spanish:Reversed	9.5047	10.6210	4.3967	<0.0001
Smooth item (Compound)	93.0669	111.0000	6.6982	<0.0001
Smooth trial by participant	141.1186	267.0000	8.1540	<0.0001
Smooth time by participant	186.7179	266.0000	4.4254	<0.0001

*Treatment coding was used for the six-level factor for the interaction of L1 by Word Order, with English Licit as reference level*.

The second half of Table 6 describes the thin plate regression spline smooths (first six rows) for the development of the amplitude over time, the nonlinear interaction of the compound's constituent frequency (second six rows), and the random-effect structure in the model (last three rows)^11^. The column labeled edf presents the *effective degrees of freedom*: smooths with higher edf tend to be more wiggly. The first smooth, for English in the licit condition, presents the development of the amplitude over time for the corresponding subset of the data. The next 5 rows evaluate difference curves with respect to the English licit condition. The summary indicates that there are significant differences between English licit and the other combinations of Group and Word Order, with the exception of Spanish in the reversed Word Order. As observed above for Compound Frequency, the Spanish in the reversed condition again pattern with the English in the licit condition.

The nonlinear interaction of the constituent frequencies by OG was modeled analogously, with a tensor smooth for English Licit, and difference smooths for the other levels of OG. As can be read of Table 6, all difference smooths reached significance.

To understand what the spline and tensor smooths represent, visualization is essential. Although visualization of the present model is straightforward, it pitches the Spanish and German, as well as the English reversed condition against the English Licit condition. Given that we have established the presence of many significant differences with English compounds in their normal word order as read by native speakers of English, we proceed with visualization based on the same model but fitted to the individual languages, contrasting the licit condition with the reversed condition (the output models are not presented in the text nor tables).

**Figure 4:**
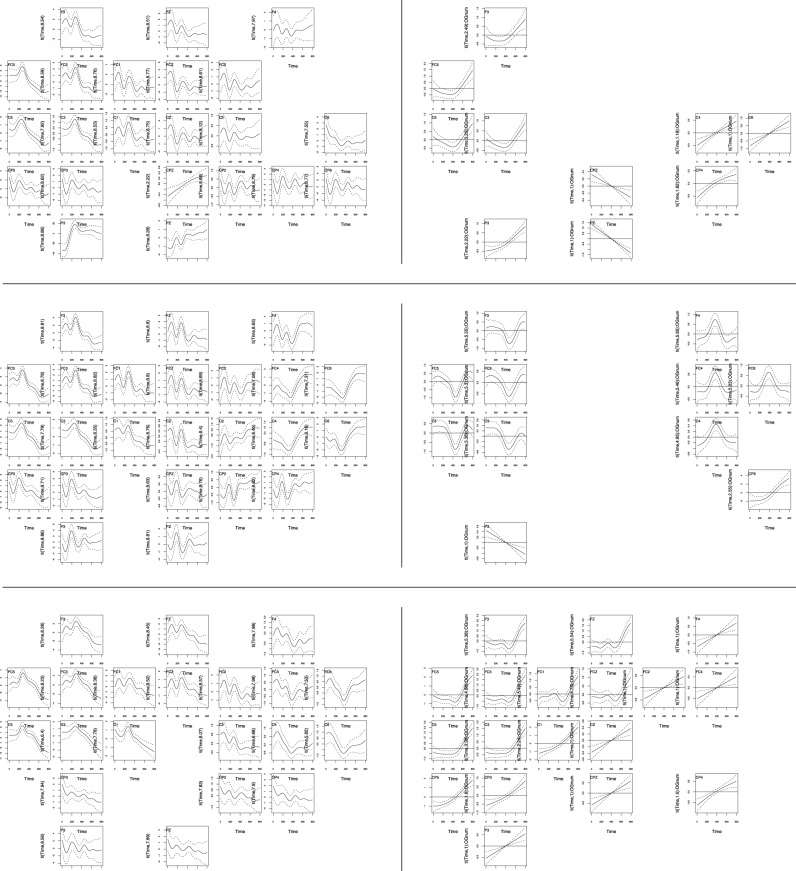
**The three-way interaction of Participant Group, Word Order, and Time**. Upper row: English, middle row: German, bottom row: Spanish; left column: amplitude development over time for the **licit** constituent order, right column: the difference curve for the **reversed** word order, with confidence intervals (dotted lines). Details of individual panels can be inspected by zooming in with higher magnification.

**Figure 5:**
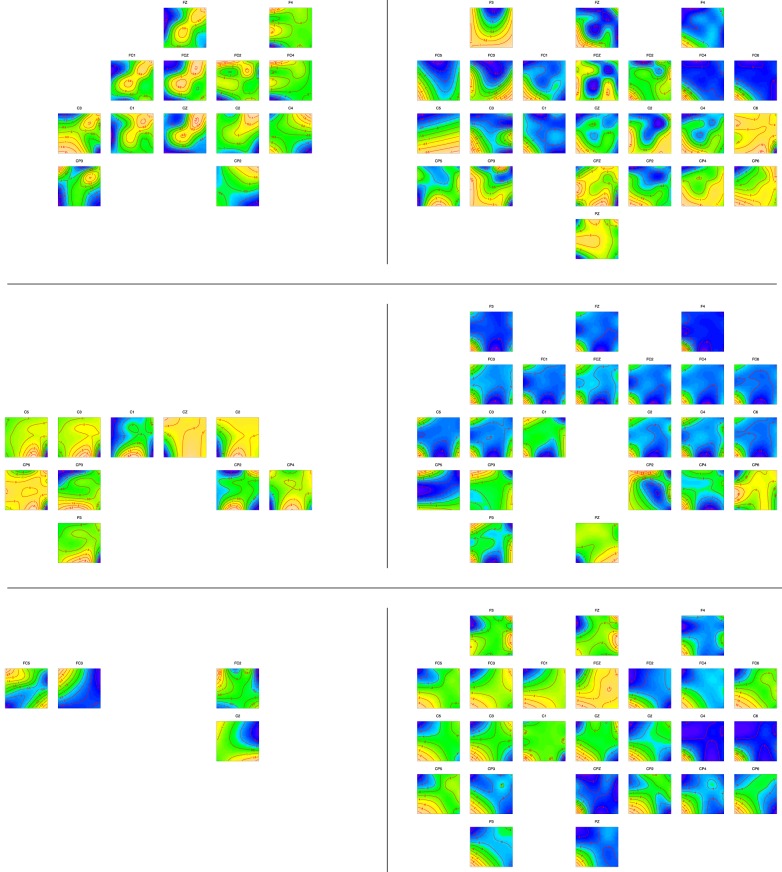
**The four-way interaction of Participant Group, Word Order, and the two constituent frequencies (horizontal: frequency of first noun, vertical: frequency of second noun)**. Upper row: English, middle row: German, bottom row: Spanish; left column: amplitude surface for the licit constituent order, right column: the difference surface for the reversed word order. Channels where there was no significant effect are not shown. Top panels present frontal channels, whereas the bottom panels show the parietal channels. Darker shades of blue indicate larger negative partial effects, whereas yellow and white denote larger positive partial effects. Details of individual panels can be inspected by zooming in with higher magnification.

Figures 4, 5 present a summary overview of the regression curves and surfaces obtained. Within each plot region (upper rows: English, middle rows: German, bottom rows: Spanish; left column: the licit condition; right column: the difference curve (or surface) for the reversed condition).

Within a plot region, panels are arranged roughly following the topography of the EEG cap, with frontal channels at the top and parietal channels at the bottom. Only those channels are shown for which the effect was significant (*p* < 0.01).

First consider the right-hand half of Figure 4, focusing on the violation condition (in which compound constituents were presented in reversed order). The upper panel of plots shows a negative inflection in the difference curve around 200–400 ms post stimulus onset at left frontal and central channels for the English speakers. A more pronounced negative inflection starting around 400 ms post stimulus onset is visible for the German speakers (center left panel), again at left frontal and central sites. Interestingly, at right frontal sites, this negative inflection reverses into a strong positivity. For the Spanish speakers, left frontal and midline channels show a reduced but still significant negative inflection in the difference curve, also starting around 400 ms. This suggests an early N400 effect for English speakers, and a standard N400 effect for the non-native groups (although delayed, as expected for non-natives—Moreno and Kutas 2005), with the strongest effect emerging for the German speakers^12^.

The N400 is traditionally considered to reflect semantic integration processes (Kutas and Federmeier, 2011), and its amplitude has been found to be larger for non-words than words (Kutas and Federmeier, 2000), including when the test items were (reversed and non-reversed) compounds (El Yagoubi et al., 2008). This ERP signature traditionally reported at more parietal electrodes, but (Voss and Federmeier, 2011) demonstrated that it can also be found in more anterior locations, as we do here.

All groups featured a significant positive peak in amplitude around 300 ms, as can be seen in the left plot regions of Figure 4. As the difference curves in the corresponding right plot regions are relatively flat for the first 300 ms, this P300 also characterized the reading of compounds with reversed word order. This effect was more pronounced for English and German speakers, and somewhat attenuated for the Spanish speakers. In all groups, this peak occurred earlier at more parietal regions in the left hemisphere, suggesting a possible spreading from parietal to frontal regions.

In the Reversed Word Order condition, the English and Spanish groups feature a significant positive inflection in amplitude at left frontal sites starting around 500 ms and rising up to the end of the time window [0-800 ms], suggesting a higher, later peak. The German group does not feature this robust pattern. Furthermore, the English and Spanish, but not the Germans, show at some right channels a linear increase in amplitude over time.

Summing up, the violation of English word order is reflected in the EEG signal by an N400 effect. For English and Spanish, a positivity around 600 ms post stimulus onset may reflect a P600 (or perhaps a P500) indexing the processing of syntactic repair or integration (Kaan, 2007). Comparing the three groups, the Spanish difference curves group together with the English difference curves, whereas the German difference curves stand apart with a stronger N400 effect at left frontal sites and, surprisingly, a P400 effect at right frontal sites.

Figure 5 presents the three-way interaction of the frequency of the first constituent (horizontal axis of each contour plot) by the frequency of the second constituent (vertical axis of each contour plot) by OG. Darker shades of blue indicate larger negative partial effects, whereas yellow and white denote larger positive partial effects.

First consider channel C3 in the upper left panel of plots of Figure 5. What this panel shows is that higher amplitudes are characteristic for compounds for which both constituent frequencies are either high (upper right corner) or low (lower left corner). Lower amplitudes are characteristic for mismatching constituent frequencies. This kind of cross-over interaction has been observed previously for the constituents of derived words in an eye-tracking study of reading (Kuperman et al., 2010), suggesting that an imbalance in constituent frequencies increases entropy, leading to an increased processing load.

This cross-over effect, which is also visible at neighboring channels (FZ, FCZ, FC1, C1, Cz, C2, C4) is present only for English readers in the licit condition. German speakers in the licit condition (center left panel) show an inverse U-shaped effect of modifier frequency for lower values of head frequency at most channels. We think this effect may be the result of the prior priming of the head constituent, which may have affected the nonnative speakers of German more than the native speakers of English. The inverse U-shaped effect may represent optimization of the response to those words which have probabilities (gauged by their corpus frequencies) that are themselves probable, i.e., in the center of the (lognormal) probability distribution. In other words, we think it is not the relative frequency of the modifier itself that predicts the amplitude, but the probability of that relative frequency.

For Spanish, significant results for the licit word order (shown in the lower left plot region) are too scattered to provide a realistic basis for interpretation.

Next consider the consequences of reversing constituent order, as shown in the right-hand half of Figure 5. For English and German (top and center panels), and more right-lateralized for Spanish (lower panel), downward adjustments of the amplitude are widespread, especially at more frontal sites in the English and German groups. We speculate that source analysis will find that these negativities reflect conflict resolution processes originating from the anterior cingulate cortex (ACC) Botvinick et al. (2001); Yeung et al. (2004): the constituents are legitimate, but their order is not, resulting in conflicting evidence for a lexicality decision. Note that the kind of “conflicts” that arise due to what is generally described as lexical competition (e.g., neighbors) is qualitatively different from the conflict arising with our experimental manipulation, which involves higher-order meaningful constituents that in half of the trials are saliently out of order.

For English, patterns across channels vary widely, with the common feature that negative effects are pervasive for high head frequencies. Since the head was primed, the appearance of the head in the inconventional initial position may have induced greater processing costs especially for higher-frequency heads.

The pattern for German (center right plot region) is much more systematic. The inverse U-shaped effect that emerged for the licit word order is negated by a U-shaped negative inflection of the EEG wave. This negative inflection is even present at many sites where no significant effect was discernable in the licit condition (see e.g., all F and FC channels). The change in polarity of the effect suggests the hypothesis that the negative, downwards, adjustments to the EEG waveform are an index of processing costs, whereas the positive (inverse U-shaped) effects in the licit condition reflect facilitated processing.

The pattern for Spanish in the reversed condition is strikingly different from that for English and German. First, the sensors in the left hemisphere reveal a pattern that bears some resemblance to the pattern for English in the licit condition, compare for instance C3 for English licit and Spanish Reversed. Compounds with constituents of similar frequency show positive inflections, whereas constituents of dissimilar frequency show negative inflections. Since the negative inflections correspond to high-entropy situations, this pattern fits nicely with the hypothesis advanced above that positive inflections reflect facilitated processing, and negative inflections, increased processing costs. The reason that the Spanish in the reversed condition pattern with the English in the licit condition is most likely to be the licitness of the reversed word order for Spanish.

Interestingly, the negative effects at many channels in the right hemisphere, as well as at more parietal channels, set the Spanish apart from English in both the licit and reversed word order conditions. We think these negativities reflect the processing invested in resolving the incongruity of the licit Spanish word order for English compounds.

## 5. Discussion

The present examination of similarities and differences between native and non-native reading of English compounds revealed the results summarized in Table 7.^13^ First, in the speeded lexical decision task (Study 1), the L2 participants' accuracy rates for compounds with licit constituent order were indistinguishable from those of native speakers of English. This indicates that the target structure of English compounds has been acquired, and that there is no representational deficit. This is unsurprising as no functional morphology is involved (Lardiere, 2008; Slabakova, 2008) and head-directionality transfer effects are expected to be short-lived (Haznedar, 1997; Unsworth, 2005).

**Table 7:**
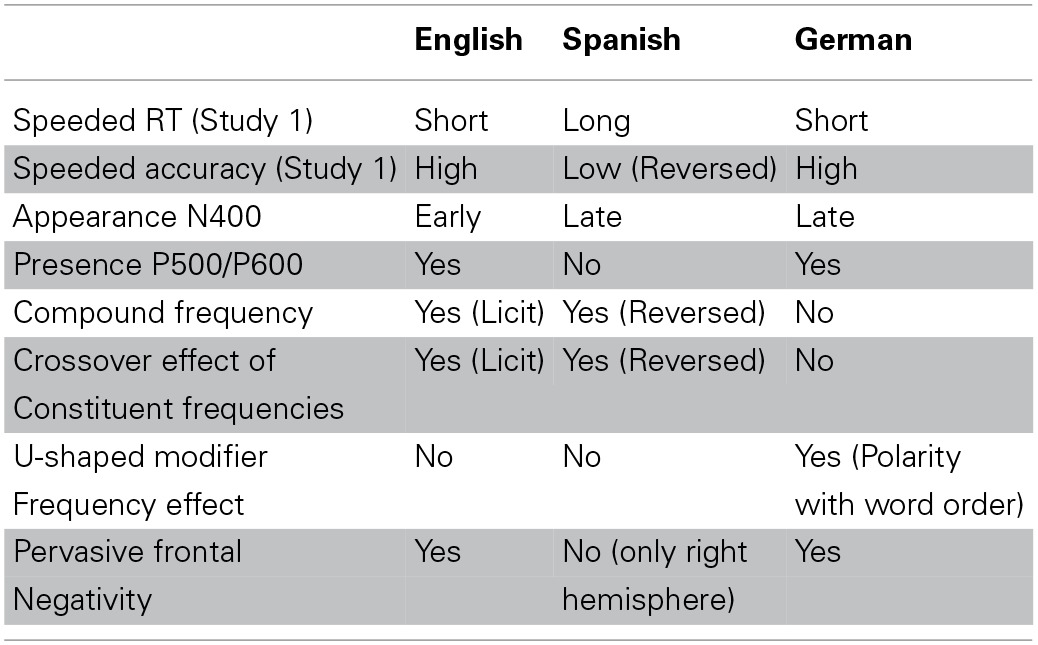
**Summary of Results**.

For compounds presented with reversed constituent order, performance dropped for all groups (except the native group in the delayed lexical decision task—Study 2). Typically, errors consisted of the over-acceptance of reversed compounds, and would be classified as ‘false alarms’ rather than “misses” in Detection Theory (Macmillan and Creelman, 2005). Whereas accuracy of German speakers was very similar to that of English speakers, the accuracy of Spanish speakers was significantly reduced under word order reversal. Furthermore, it was only for the Spanish speakers that response latencies were significantly slower than those of English native speakers (in Study 1), a result not expected according to the Interface Hypothesis (Sorace, 2011)—which predicts similar processing difficulties in the non-native language, irrespective of the properties of the L1. The slower responses of the Spanish L2 speakers suggest an interference effect from their native language: Rejecting a compound presented in reversed order requires the Spanish participants to reject what would be a licit word order in their L1. This is where they make errors, and where their responses become elongated. These results reveal the presence of L1-induced residual errors in the processing of a core grammar phenomenon.

The ERP results for Spanish fit well the presence of an L1 effect. The Spanish speakers show an effect of compound frequency, just as the English speakers, but for the reversed (i.e., their native) word order. The Spanish speakers also show a crossover effect of the constituent frequencies, as do the English speakers, again for the reversed instead of the licit word order. The compound frequency effect suggests familiarity with the onomasiological function of the compound when the constituents appear in the order appropriate for their L1. The crossover effect of the constituent frequencies is likewise conditioned on the order in the speakers' L1, and may bear witness to higher processing costs when the entropy of the probability distribution of modifier and head [as gauged by their (relative) frequencies] is high (see Kuperman et al., 2010).

The frequency effects present for the German speakers are very different from both those of English and of Spanish speakers. Their EEG signal was not predictable from compound frequency, suggesting decomposition (i.e., full parsing). Furthermore, the constituent frequency effects were different in nature, showing for modifier frequency (conditional on a low head frequency) an inverse U-shaped curve for licit word order, and a U-shaped pattern for the reversed word order. For these speakers, the violation condition is characterized by topographically pervasive negativities. This suggests that German speakers were especially sensitive to the word order violation in English, which also violates the expected word order in German. Support for this hightened sensitivity comes from the N400 effect for this group of speakers, which is characterized by a well-defined narrow large downward inflection for the reversed compounds. Of course, the speakers of the other two languages must also have been aware of the violations, as indicated by their increased error rates and longer response latencies. Nevertheless, the N400 effects for the English and Spanish speakers are not as pronounced as for the German speakers. A final difference between the German speakers and the other two language groups, for which we have no explanation, is the absence of a clear positivity starting around 600 ms post stimulus onset (possibly a P500 or a P600 effect indexing reanalysis and repair), and the presence of a positive inflection around 400 ms post stimulus onset at channels at right frontal sites, the mirror image of the N400 effect.

An alternative interpretation for the negativity observed around 400 ms post-stimulus onset in the present study is that it reflects the left anterior negativity (LAN) component which is assumed to index integration of morphosyntactic information (Friederici, 1995, 2001; Steinhauer et al., 2009)^14^. In fact, the scalp distribution of the observed component (anterior and predominantly left) does align with LAN. The LAN has been shown to be elicited by subject verb agreement violations (but not by number or gender violations between an antecedent and a reflexive pronoun—Osterhout and Mobley 1995), grammatical gender violation (Gunter et al., 2000), and pronoun case and verb agreement errors (Coulson et al., 1998). Though the LAN component is typically observed in studies with sentence stimuli, it is possible to interpret our findings as a LAN if we assume that the processing and violations in the compounds used in the present study are morpho-syntactic rather than semantic in nature. Assuming that the anterior negativity is LAN, rather than N400, and indexes morpho-syntactic processing rather than semantic processing, the results are consistent with El Yagoubi et al. (2008), who found a more negative peak in the left anterior negativity (LAN) component for compounds than for noncompounds. Arcara et al. (2014) further reported an enhanced LAN in head-final compounds in Italian, which they argue indicates they are decomposed differently to head-initial compounds (the latter being seemingly processed as syntactic-like structures rather than morphological complex words). LAN modulation has also been noted in two ERP papers on German compound processing (Koester et al., 2004, 2007). These researchers argued for compound decomposition during comprehension providing evidence against full-listing models and in favor of decomposition or dual-route models of compound processing.

The P300 effect that we observed for all participant groups in both word order conditions could be linked to the binary decision (licit/illicit) the participants had to make regarding the stimuli (Donchin and Coles, 1988; Barber and Carreiras, 2005). Thus, regardless of whether the stimuli were licit or illicit, participants had to attend and indicate their decision: the P300 here could be interpreted as indexing attention associated with language processing. Several authors have proposed that P300 activity is related to subsequent P600 activity for reanalysis and repair processes (e.g., Friederici, 1995).

All groups were sensitive to the probabilities of the modifier and head constituents. This challenges the claim of Silva-Corvalan and Clahsen (2008) that non-native speakers would rely on whole-word processing without understanding the constituents, but is consistent with a syntactic analysis of noun-noun compounds. Our results suggest that lexically transparent NNCs with low frequencies are processed combinatorially by (advanced) non-native speakers, as they are by native speakers (MacGregor and Shtyrov, 2013). Our findings are also consistent with the conjoint effects of both whole-word and constituent probabilities in the eye-tracking record, as early as first fixation durations (see, e.g., Kuperman et al., 2008, 2009; Miwa et al., 2014, for English, Finnish, and Japanese respectively). The importance of the constituents for non-native speakers is reminiscent of the decompositional eye-movement patterns of less-proficient readers reported by (Kuperman and Van Dyke, 2011).

Our study confirms the importance of the Third Factor (Chomsky, 2005) in L2 research: it suggests that processing effects can be induced by properties of the L1 that cannot be fully inhibited during L2 processing, in spite of acquisition of the target representation. In terms of Detection Theory (Macmillan and Creelman, 2005), this predicts that false alarms (i.e., accepting an illicit structure) will persist when misses (i.e., failing to accept a licit structure) have dropped to non-significant levels. It might be that domain-general inhibition is required to suppress L1 interferences in L2 processing, in the same way as it is recruited for language switching (de Bruin et al., 2014), in which case a corrleation would be expected between the rate of false alarms and inhibition abilities (all other things being equal).

Methodologically, the insights gleaned from the EEG amplitudes would not have been possible without generalized additive mixed models. At the same time, we believe we are only seeing the tip of the iceberg. For instance, the model can be improved by allowing the interaction of the constituent frequencies by group and constituent order to vary with time, using five-way tensor product smooths. Two considerations have withheld us from following up on such considerably more complex models. First, without specific hypotheses as a guide, interpretation becomes extremely difficult. Second, we are concerned that with a relative small number of compounds (120), overfitting might become an issue. For future research specifically addressing the development over time of constituent (and whole-compound) frequency effects, we recommend regression designs with substantially larger numbers of compounds. Replication studies will be essential for boosting confidence in the nonlinear effects revealed by the GAMMs.

## Author contributions

Cecile De Cat: The first author conceived the project and was substantially involved in all aspects of its design and realization (except for data collection), as well as in the analysis and interpretation, and the drafting and revision of the manuscript. Ekaterini Klepousniotou: The second author contributed substantially to the design and realization, oversaw the data collection and initial data preparation, contributed to the interpretation of the results and critically revised the manuscript. R. Harald Baayen: The third author led and substantially contributed to the analysis of the ERP data and its interpretation, and contributed substantially to the drafting of the relevant sections and conclusions. All authors are responsible for final approval of the version to be published and agree to be accountable for all the aspects of the work in ensuring that questions related to the accuracy or integrity of any part of the work are appropriately investigated and resolved.

## Funding

The third author was supported by an Alexander von Humboldt research chair awarded by the Alexander von Humboldt foundation, and the first author was supported by a British Academy Skills Acquisition award (SQ120066) and by the Leeds Humanities Research Institute.

### Conflict of interest statement

The authors declare that the research was conducted in the absence of any commercial or financial relationships that could be construed as a potential conflict of interest.

## Figures and Tables

**Figure A1:**
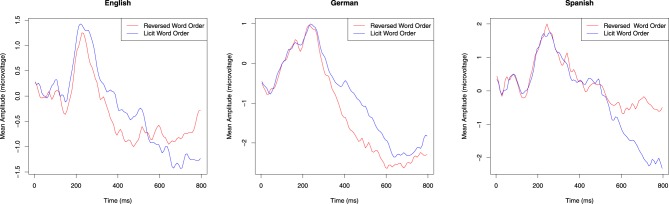
**Grand average ERP for the raw data at electrode C3, by participant group and Word Order condition**.

**Figure A2:**
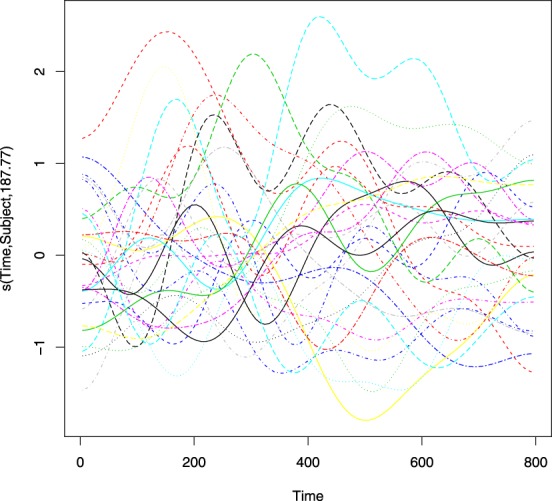
**Average amplitude (μV) over time (ms) by participant at electrode C3**. Each line corresponds to a participant.

## Footnotes

^1^Cross-linguistically, head directionality transfer effects in L2 acquisition have been found to be very short-lived, both in child and in adult learners—see e.g., Haznedar (1997); Unsworth (2005).

^2^Each compound was presented one by one in licit and reversed order (in randomized order), and participants were asked to rate them by choosing one of the following options: perfectly ok—rare but ok—strange but ok—a bit too strange—very strange—completely bad. These ratings were converted into a numeric score expressed as a percentage. The frequency of the intended compound did not predict its reversibility (Pearson's product-moment correlation: ρ = 0.14. *t* = 1.5766, df = 115, *p* = 0.1176).

^3^The stimuli and their frequency statistics are given in Tables A1, A2 in the Appendix. The length of the stimuli ranged from 7 to 18 characters (mean:11.9).

^4^Table A3 in the Appendix gives a summary of the random effects for this model.

^5^Table A4 in the Appendix gives a summary of the random effects for this model. Table A5 in the Appendix give the mean reaction times by participant group and condition.

^6^The average visual angle subtended was 5.7°: the stimuli extended approximately 2.8° to the left and right of the center of the screen.

^7^Table A6 in the Appendix gives a summary of the random effects for this model.

^8^The grand average ERPs for the raw data can be found in Figure A1 in the Appendix.

^9^Using *post-hoc* correlation analyses, (Tanner et al., 2013) found that grand mean waveforms showing a biphasic N400 + P600 response in fact concealed a more complex pattern, in which most individuals showed either an N400 or a P600, but not both.

^10^31 other models were fitted, one per chanel. Each single-chanel analysis was carried out on 609,500 data points. The main results of these models are summarized by means of Figures 4, 5. Patterns that show geographical consistency across neighboring channels are the ones we have most confidence in. We focus on C3 in the model presentation, as a representative chanel for the effects of interest in our study. A baseline period was not included in the figures, because the pre-target window is one for which differential effects are expected, as different primes are presented. At −100 ms before the target word, the prime is still being read (−100 to −50 ms, followed by 50 ms of mask). Baselining has been carried out to nullify intercept shifts due to the prime, but we do NOT expect the same profile across conditions, because the primes are different, and related to the compounds in different ways.

^11^The random effect for participant over time is plotted in Figure A2 in the Appendix.

^12^But see Discussion for an alternative explanation of the observed negative inflection as LAN.

^13^With a high value for the ρ parameter, our analyses are conservative. Furthermore, with a Bonferroni correction for 32 channels by 27 coefficients or smooth terms, any term in Table 6 for which *p* < 0.0001 is reasonably well supported. Nevertheless, with only 10 speakers for each group, only a replication study can reveal how robust the regression curves and regression surfaces actually are.

^14^Thanks to an anonymous reviewer for suggesting this.

## Appendix

**Table A1:** **Stimuli (in licit word order)**.

Adult jail	Air missile	Alcohol licence
Army depot	Ash cloud	Baby lotion
Bacon rind	Banana pancake	Bath mat
Beach ball	Bicycle bell	Bike grease
Bird virus	Blackcurrant jelly	Bread knife
Bronze manequin	Calf liver	Camp chair
Candle wick	Canine tooth	Cannabis resin
Car pollution	Cartoon series	Cattle grid
Cell nucleus	Cement block	Champagne froth
Cherry jar	Chestnut mash	Chicken leg
Church minister	Cigarette smell	Clay doll
Clothes peg	Coal dust	Coconut tree
Council leaflet	Country produce	Crime trend
Custard layer	Diary extract	Dog basket
Dress pattern	Duck poo	Ferry fume
Finance consultant	Floor tile	Flour dough
Flower petal	Flu injection	Freezer magnet
Garlic clove	Geography essay	Gold broach
Granola bar	Gravel path	Gym bag
Holiday souvenir	Home remedy	Hydrogen bubble
Ice sculpture	Ink stain	Jungle Beast
Kitchen utensil	Lace edge	Lamb kidney
Lemon zest	Lightning strike	Limestone rock
Maple leaf	Marble inkpot	Metal gate
Milk powder	Mountain goat	Music certificate
Nappy rash	Nettle juice	Nose drop
Ocean navigation	Papaya smoothie	Paper hat
Party outfit	Pen lid	Pet odour
Phone socket	Pig enclosure	Piston shaft
Plastic obstacle	Protein ingredient	Radio source
Rat poison	Rubber glove	Ruby pendant
Safety rule	Sandwich snack	Sea fish
Silver ring	Sleeve patch	Soup dish
Space debris	Sport injury	Steel rod
Stone chisel	Sun deck	Sweat band
Tea cart	Teak partition	Team mascot
Throat tablet	Timber fence	Tobacco product
Traffic noise	Travel kettle	Turtle shell
Vanilla cream	War troop	Wood preservative

**Table A2:** **Frequency statistics for the stimuli (in licit word order)**.

	**Mean**	**SD**	**Median**	**Min**	**Max**
Compound	359.52	640.78	96	0	3300
First constituent	279297.19	420859.13	120738	0	2759265
Second constituent	278976.08	421000.19	116003	0	2759265

**Table A3:** **Random effects from the logistic mixed-effects regression model fitted to the accuracy data of Study 1**.

**Groups**	**Name**	**Variance**	**Std.Dev**.	**Corr**
Target	(Intercept)	0.7058	0.8401	
Subject	(Intercept)	0.2380	0.4879	
	Word.Order:Reversed	0.1831	0.4279	−0.08

*Inclusion of by-target random slopes for group resulted in an overspecified model, and therefore was removed*.

**Table A4:** **Random effects from the logistic mixed-effects regression model fitted to the reaction time data of Study 1**.

**Groups**	**Name**	**Variance**	**Std.Dev**.	**Corr**	
Target	(Intercept)	0.006527	0.08079		
	L1German	0.005625	0.07500	0.12	
	L1Spanish	0.011570	0.10756	0.43	0.75
Subject	(Intercept)	0.025052	0.15828		
	Word.Order:Reversed	0.002446	0.04946	−0.28

**Table A5:** **Mean Reaction Times (in ms) by Participant Group and Word Order condition in the speeded lexical decision task (Study 1)**.

	**English**	**German**	**Spanish**
Licit word order	877.07	1473.11	1566.14
Reversed word order	973.99	1588.83	1625.23

**Table A6:** **Random effects from the logistic mixed-effects regression model fitted to the reaction time data of Study 2**.

**Groups**	**Name**	**Variance**	**Std.Dev**.	**Corr**	
Target	(Intercept)	0.9445	0.9718		
	L1German	0.7516	0.8669	−0.44	
	L1Spanish	0.7443	0.8627	−0.29	0.75
Participant	(Intercept)	0.5815	0.7626		
	Word.OrderReversed	0.6033	0.7767	−0.71	

## References

[B1] Arcara G. Marelli M. Buodo G. Mondini S. (2014). Compound headedness in the mental lexicon: an event-related potential study. Cogn. Neuropsychol. 31, 164–183. 10.1080/02643294.2013.847076 24168167

[B2] Barber H. Carreiras M. (2005). Grammatical gender and number agreement in spanish: an erp comparison. J. Cogn. Neurosci. 17. 137–153. 10.1162/0898929052880101 15701245

[B3] Bates D. Maechler M. Bolker B. Walker S. (2013). lme4: Linear Mixed-Effects Models Using Eigen and S4. R package version 1.0-4.

[B4] Bertram R. Hyönä J. Pollatsek A. (2004). Morphological parsing and the use of segmentation cues in reading Finnish compounds. J. Mem. Lang. 51, 325–345 10.1016/j.jml.2004.06.005

[B5] Botvinick M. Braver T. Carter C. Barch D. Cohen J. (2001). Evaluating the demand for control: anterior cingulate cortex and crosstalk monitoring. Psychol. Rev. 108, 624–652 10.1037/0033-295X.108.3.624 11488380

[B6] Briggs G. Nebes R. (1975). Patterns of hand preference in a student population. Cortex 11, 230–238. 10.1016/S0010-9452(75)80005-0 1204363

[B7] Butterworth B. (1983). Lexical representation, in Language Production, ed Butterworth B. (San Diego, CA: Academic Press), 257–294.

[B8] Chomsky N. (2005). Three factors in language design. Linguist. Inq. 36, 1–22 10.1162/0024389052993655

[B9] Clahsen H. Balkhair L. Schutter J.-S. Cunnings I. (2013). The time course of morphological processing in a second language. Second Lang. Res. 29, 7–31 10.1177/0267658312464970

[B10] Coulson S. King J. Kutas M. (1998). Expect the unexpected: event-related brain response to morphosyntactic violations. Lang. Cogn. Process. 13, 21–58 10.1080/016909698386582

[B11] de Bruin A. Roelofs A. Dijkstra T. FitzPatrick I. (2014). Domain-general inhibition areas of the brain are involved in language switching: fmri evidence from trilingual speakers. NeuroImage 90, 348–359. 10.1016/j.neuroimage.2013.12.049 24384153

[B12] Donchin E. Coles M. (1988). Is the p300 component a manifestation of context updating? Behav. Brain Sci. 11, 357–374. 10.1017/S0140525X0005802722974337

[B13] El Yagoubi R. Chiarelli V. Mondini S. Perrone G. Danieli M. Semenza C. (2008). Neural correlates of Italian nominal compounds and potential impacts of headedness effect: an ERP study. Cogn. Neuropsychol. 25, 559–581. 10.1080/02643290801900941 19086202

[B14] Friederici A. D. (1995). The time course of syntactic activation during language processing: a model based on neuropsychological and neurophysiological data. Brain Lang. 50, 259–281. 10.1006/brln.1995.1048 7583190

[B15] Friederici A. D. (2001). Event-related brain potentials and aphasia, in Handbook of Neuropsychology, 2nd Edn., Vol. 3, eds Boller F. Grafman J. (Amsterdam: Elsevier Science), 353–373.

[B16] Gagné C. L. Spalding T. L. (2014). Conceptual composition: the role of relational competition in the comprehension of modifier-noun phrases and noun-noun compounds. Psychol. Learn. Motiv. 59, 97–130 10.1016/B978-0-12-407187-2.00003-4

[B17] Gunter T. Friederici A. Schriefers H. (2000). Syntactic gender and semantic expectancy: erps reveal early autonomy and late interaction. J. Cogn. Neurosci. 12, 556–568. 10.1162/089892900562336 10936910

[B18] Haznedar B. (1997). Child Second Language Acquisition of English: A longitudinal Case Study of a Turkish-Speaking Child. Doctoral dissertation, Durham University, Durham.

[B19] Hyönä J. Pollatsek A. (1998). Reading finnish compound words: eye fixations are affected by component morphemes. J. Exp. Psychol. Hum. Percept. Perform. 24, 1612–1627. 10.1037/0096-1523.24.6.1612 9861713

[B20] Jarema G. (2006). Compound representation and processing: a cross-language perspective, in The Representation and Processing of Compound Words, eds Libben G. Jarema G. (Oxford: OUP), 45–70.

[B21] Jarema G. Busson C. Nikolova R. Tsapkini K. Libben G. (1999). Processing compounds: a cross-linguistic study. Brain Lang. 68, 362–369. 10.1006/brln.1999.2088 10433782

[B22] Juhasz B. Starr M. Inhoff A. Placke L. (2003). The effects of morphology on the processing of compound words: evidence from lexical decision, naming, and eye fixations. Br. J. Psychol. 94, 223–244. 10.1348/000712603321661903 12803817

[B23] Kaan E. (2007). Event-related potentials and language processing. a brief introduction. Lang. Linguist. Compass 1, 571–591. 10.1111/j.1749-818X.2007.00037.x23177656

[B24] Koester D. Gunter T. C. Wagner S. (2007). The morphosyntactic decomposition and semantic composition of german compound words investigated by erps. Brain Lang. 102, 64–79. 10.1016/j.bandl.2006.09.003 17055044

[B25] Koester D. Gunter T. C. Wagner S. Friederici A. D. (2004). Morphosyntax, prosody, and linking elements: the auditory processing of german nominal compounds. J. Cogn. Neurosci. 16, 1647–1668. 10.1162/0898929042568541 15601526

[B26] Kroll J. F. Michael E. Tokowicz N. Dufour R. (2002). The development of lexical fluency in a second language. Second Lang. Res. 18, 137–171 10.1191/0267658302sr201oa

[B27] Krott A. Gagne C. L. Nicoladis E. (2010). Children's preference for has and located relations: a word learning bias for noun-noun compounds. J. Child Lang. 37, 373–394. 10.1017/S0305000909009593 19490749

[B28] Kryuchkova T. Tucker B. V. Wurm L. Baayen R. H. (2012). Danger and usefulness in auditory lexical processing: evidence from electroencephalography. Brain Lang. 122, 81–91. 10.1016/j.bandl.2012.05.005 22726720

[B29] Kuperman V. Bertram R. Baayen R. H. (2008). Morphological dynamics in compound processing. Lang. Cogn. Process. 23, 1089–1132 10.1080/01690960802193688

[B30] Kuperman V. Bertram R. Baayen R. H. (2010). Processing trade-offs in the reading of Dutch derived words. J. Mem. Lang. 62, 83–97 10.1016/j.jml.2009.10.001

[B31] Kuperman V. Schreuder R. Bertram R. Baayen R. H. (2009). Reading of multimorphemic Dutch compounds: towards a multiple route model of lexical processing. J. Exp. Psychol. Hum. Percept. Perform. 35, 876–895. 10.1037/a0013484 19485697

[B32] Kuperman V. Van Dyke J. (2011). Effects of individual differences in verbal skills on eye-movement patterns duing sentence reading. J. Mem. Lang. 65, 42–73. 10.1016/j.jml.2011.03.002 21709808 PMC3119501

[B33] Kutas M. Federmeier K. D. (2000). Electrophysiology reveals semantic memory use in language comprehension. Trends Cogn. Sci. 4, 462–470. 10.1016/S1364-6613(00)01560-6 11115760

[B34] Kutas M. Federmeier K. D. (2011). Thirty years and counting: finding meaning in the n400 component of the event-related brain potential (erp). Annu. Rev. Psychol. 62, 621–647. 10.1146/annurev.psych.093008.131123 20809790 PMC4052444

[B35] Lardiere D. (2008). Feature assembly in second language acquisition, in The Role of Formal Features in Second Language Acquisition, eds Liceras J. Zobl H. Goodluck H. (New York, NY: Lawrence Erlbaum Associates), 106–140.

[B36] Libben G. (1998). Semantic transparency in the processing of compounds. Brain Lang. 61, 30–44. 10.1006/brln.1997.1876 9448929

[B37] Libben G. (2006). Why study compound processing? An overview of the issues, in The Representation and Processing of Compound Words, eds Libben G. Jarema G. (Oxford: OUP), 1–22.

[B38] Libben G. Jarema G. (eds.). (2006). The Representation and Processing of Compound Words. Oxford: OUP.

[B39] Lieber R. Štekauer P. (eds.). (2009). The Oxford Handbook of Compounding. Oxford: Oxford University Press.

[B40] MacGregor L. J. Shtyrov Y. (2013). Multiple routes for compound word processing in the brain: evidence from eeg. Brain Lang. 126, 217–229. 10.1016/j.bandl.2013.04.002 23800711 PMC3730057

[B41] Macmillan N. Creelman C. (2005). Detection Theory: A User's Guide. Mahwah, NH: Lawrence Erlbaum Associates.

[B42] Marelli M. Crepaldi D. Luzzatti C. (2009). Head position and the mental representation of italian nominal compounds. Ment. Lexicon 4, 430–455. 10.1075/ml.4.3.05mar24313592

[B43] Marelli M. Zonca G. Contardi A. Luzzatti C. (2014). The representation of compound headedness in the mental lexicon: a picture naming study in aphasia. Cogn. Neuropsychol. 31, 26–39. 10.1080/02643294.2013.860024 24313592

[B44] Meyer R. (1993). Compound Comprehension in Isolation and in Context. The Contribution of Conceptual and Discourse Knowledge to the Comprehension of German Novel Noun-Noun Compounds. Berlin: Walter de Gruyter.

[B45] Miwa K. Libben G. Dijkstra T. Baayen H. (2014). The time-course of lexical activation in japanese morphographic word recognitin: evidence for a character-driven processing model. Q. J. Exp. Psychol. 67, 79–113. 10.1080/17470218.2013.790910 23713954

[B46] Moreno E. M. Kutas M. (2005). Processing semantic anomalies in two languages: an electrophysiological exploration in both languages of spanish-english bilinguals. Brain Res. Cogn. Brain Res. 22, 205–220. 10.1016/j.cogbrainres.2004.08.010 15653294

[B47] Nicoladis E. Yin H. (2002). The role of frequency in acquisition of english and chinese compounds by bilingual children, in Proceedings of the Annual Boston University Conference on Language Development, Vol. 26, (Somerville, MA: Cascadilla Press), 441–452.

[B48] Osterhout L. Mobley L. A. (1995). Event-related brain potentials elicited by failure to agree. J. Mem. Lang. 34, 739–773 10.1006/jmla.1995.1033

[B49] Otten L. Rugg M. (2005). Interpreting event-related brain potentials, in Event-Related Potentials: A Methods Handbook, ed Handy T. (Cambridge, MA: MIT Press), 3–17.

[B50] Piera C. (1995). On compounding in english and spanish, in Evolution and Revolution in Linguistic Theory, eds Campos H. Kempchinsky P. (Washington, DC: Georgetown University Press), 301–315.

[B51] Pollatsek A. Hyönä J. Bertram R. (2000). The role of morphological constituents in reading Finnish compound words. J. Exp. Psychol. Hum. Percept. Perform. 26, 820–833. 10.1037/0096-1523.26.2.820 10811178

[B52] Sandra D. (1990). On the representation and processing of compound words: automatic access to constituent morphemes does not occur. Q. J. Exp. Psychol. 42A, 529–567 10.1080/14640749008401236

[B53] Semenza C. Luzzatti C. (2014). Combining words in the brain: the processing of compound words.introduction to the special issue. Cogn. Neuropsychol. 31, 1–7. 10.1080/02643294.2014.898922 24784361

[B54] Semenza C. Luzzatti C. Carabelli S. (1997). Morphological represntation of compound nouns: a study on Italian aphasic patients. J. Neurolinguist. 10, 33–43. 10.1016/S0911-6044(96)00019-X15172563

[B55] Sharbrough F. Chatrian G. Lesser R. Luders H. Nuwer M. Picton T. (1991). American electroencephalographic society guidelines for standard electrode position nomenclature. J. Clin. Neurophysiol. 8, 200–202. 10.1097/00004691-199104000-00007 2050819

[B56] Silva-Corvalan C. Clahsen H. (2008). Morphologically complex words in l1 and l2 processing: evidence from masked priming experiments in english. Bilingualism 11, 245–260 10.1017/S1366728908003404

[B57] Slabakova R. (2008). Meaning in the Second Language. Studies on Language Acquisition 34. Berlin: Mouton de Gruyter.

[B58] Sorace A. (2011). Pinning down the concept of “interface” in bilingualism. Linguist. Approaches Bilingualism 1, 1–33 10.1075/lab.1.1.01sor

[B59] Steinhauer K. White E. J. Drury J. E. (2009). Temporal dynamics of late second language acquisition: evidence from event-related brain potentials. Second Lang. Res. 25, 13–41 10.1177/0267658308098995

[B60] Tanner D. Inoue K. Osterhout L. (2013). Brain-based individual differences in online L2 grammatical comprehension. Bilingualism 17, 277–293 10.1017/S1366728913000370

[B61] Tremblay A. Baayen R. H. (2010). Holistic processing of regular four-word sequences: a behavioral and ERP study of the effects of structure, frequency, and probability on immediate free recall, in Perspectives on Formulaic Language: Acquisition and Communication, ed Wood D. (London: The Continuum International Publishing Group), 151–173.

[B62] Tremblay A. Newman A. (2015). Modelling non-linear relationships in ERP data using mixed-effects Regression with R examples. Psychophysiology 52, 124–139. 10.1111/psyp.12299 25132114

[B63] Unsworth S. (2005). Child L2, Adult L2, Child L1: Differences and Similarities. A Study on the Acquisition of Direct Object Scrambling in Dutch. Utrecht: LOT.

[B64] Voss J. Federmeier K. D. (2011). Fn400 potentials are functionally identical to n400 potentials and reflect semantic processing during recognition testing. Psychophysiology 48, 532–546. 10.1111/j.1469-8986.2010.01085.x 20701709 PMC2982896

[B65] Wood S. (2004). Stable and efficient multiple smoothing parameter estimation for generalized additive models. J. Am. Stat. Assoc. 99, 673–686 10.1198/016214504000000980

[B66] Wood S. (2006). Generalised Additive Models: An Introduction with R. Boca Raton, FL: Chapman and Hall/CRC.

[B67] Yeung N. Botvinick M. M. Cohen J. D. (2004). The neural basis of error detection: conflict monitoring and the error-related negativity. Psychol. Rev. 111:931. 10.1037/0033-295X.111.4.931 15482068

[B68] Zhang J. I. E. Anderson R. C. Wang Q. Packard J. Wu X. Tang S. (2012). Insight into the structure of compound words among speakers of chinese and english. Appl. Psycholinguist. 33, 753–779 10.1017/S0142716411000555

[B69] Zipser K. (2013). Proto-language, phrase structure and nominal compounds. Which of them fit together?, in Poster presented at ICL 2013 (Geneva).

